# Formation of distinct prion protein amyloid fibrils under identical experimental conditions

**DOI:** 10.1038/s41598-020-61663-2

**Published:** 2020-03-12

**Authors:** Mantas Ziaunys, Tomas Sneideris, Vytautas Smirnovas

**Affiliations:** Institute of Biotechnology, Life Sciences Center, Vilnius University, Vilnius, Lithuania

**Keywords:** Prions, Prions, Protein aggregation

## Abstract

Protein aggregation into amyloid fibrils is linked to multiple neurodegenerative disorders, such as Alzheimer’s, Parkinson’s or Creutzfeldt-Jakob disease. A better understanding of the way these aggregates form is vital for the development of drugs. A large detriment to amyloid research is the ability of amyloidogenic proteins to spontaneously aggregate into multiple structurally distinct fibrils (strains) with different stability and seeding properties. In this work we show that prion proteins are capable of forming more than one type of fibril under the exact same conditions by assessing their Thioflavin T (ThT) binding ability, morphology, secondary structure, stability and seeding potential.

## Introduction

Prion proteins are cell surface glycoproteins, most widely known for their link to transmissible spongiform encephalopathies, such as Scrapie, Creutzfeldt-Jakob disease and Gerstmann-Straussler-Scheinker syndrome^1–4^. In their native form, prion proteins exist in a mostly alpha-helical conformation (PrP^C^)^5^, however, conformational changes due to various environmental factors may induce the formation of an insoluble, β-sheet rich structure (PrP^Sc^)^6^. PrP^Sc^ acts as a template and an aggregation center for further fibril growth by incorporating monomers and changing them to the PrP^Sc^ form^7,8^. Such aggregation eventually leads to higher oligomers, protofibrils and eventually to fully formed amyloid fibrils^9^.

Amyloid fibrils are highly structured, densely packed protein aggregates^10^ which have been found in amyloid-plaques in patients with neurodegenerative disorders^11^. Their cytotoxic effect was also shown on numerous occasions with both *in vitro*^12–14^ and *in vivo*^15,16^ experiments. It has been observed that prion protein amyloid fibrils can exist in multiple distinct structural conformations^17–19^. *In vitro*, different strains can be formed based on the environmental conditions in which the aggregates are generated, such as sample agitation^20,21^, pH^22^, denaturant^23,24^, and salt concentration^25^. While the process of how and why a protein with the exact same sequence can possess multiple different fibrillar structures is of great interest and is being widely examined^26–28^, it does cause problems when analyzing and comparing data. The different strains have distinct morphologies^29,30^ and secondary structures^22,31^, replication rates^32^, stability in denaturants^28,33^. This inevitably results in data obtained from heterogeneous mixtures^34,35^. There has been an ongoing effort to not only differentiate^36^, but to purify strains of prion protein fibrils^37^. However, as of yet, single strain purification is still difficult^38^.

A commonly used method for amyloid fibril detection is a ThT assay, in which the fluorescent dye molecules specifically bind to beta-sheet grooves on the fibril’s surface, causing a red-shift in their excitation/emission spectra, as well as a large increase in fluorescence intensity^39^. ThT has been shown to have distinct binding capacity on different types of fibrils, most likely due to the structure and quantity of possible binding sites^40–42^. This specific affinity could potentially be used as a quick primary way of differentiating between samples that contain differently structured aggregates.

In this work we generated a range of mouse prion protein (MoPrP) fibril samples using the exact same conditions and attempted to separate the formed aggregate types by a ThT assay and further examine the structure, stability and seeding ability of the distinct samples. We show that under the selected conditions, there appear to be at least two mouse prion protein fibril types with different structural and seeding properties.

## Methods

### Amyloid fibril formation

Mouse recombinant prion protein C-terminal fragment (MoPrP89-230) was purified as described previously^43^. In short, the protein containing a His-tag was expressed in *E. coli*, inclusion bodies were dissolved in a 6 M guanidine hydrochloride (GuHCl) solution and the protein was loaded onto an immobilized metal affinity chromatography nickel column, refolded and eluted with a 700 mM imidazole solution. The purified protein was dialyzed into 10 mM sodium acetate (pH 4) buffer at 4 °C, filtered, concentrated to 3 mg/ml and stored at −80 °C. Typically about 100 mg of the protein is purified in a single batch. The stock solution was mixed with 50 mM sodium phosphate buffers (pH 6.0) with or without 6 M GuHCl to a final 0.5 mg/ml protein and 2 M GuHCl concentration. The solution was then evenly distributed to 20 test tubes (Fisher, #15432545) (1 ml solution per tube). In order to confirm that the distribution process does not yield samples containing different types and amounts of oligomeric species or aggregates, light scattering and ThT fluorescence assays were carried out with aliquots from 20 test tubes (Fig. S1). The tubes were placed in a shaker incubator (IKA KS 4000i) parallel to the shaker’s surface and incubated at 37 °C with constant shaking at 220 RPM for 72 hours to ensure the aggregation reaction is complete without having to periodically measure the ThT fluorescence of aliquots from each sample (typical time required is 20–30 hours (Fig. S2)). After fibril formation, an aliquot of each sample was taken for atomic force microscopy (AFM) examination, while the remaining samples were sonicated for 60 s (Bandelin Sonopuls 3100 ultrasonic homogenizer, MS-72 tip, 20% sonication strength) prior to further experiments.

### ThT fluorescence assays

99 µL aliquots of each sonicated fibril sample were mixed with 1 µL of 10 mM stock ThT solution to a final ThT concentration of 100 µM. The 100 µL sample fluorescence intensities were measured using a Varian Cary Eclipse spectrophotometer using 440 nm excitation and 460–500 nm emission range (excitation slit width – 10 nm, emission slit – 5 nm). Fluorescence intensities for each sample were the average of three measurements.

For the ThT affinity assay, 25 µL of each sample was diluted to 100 µL using a range of different concentration ThT solutions (containing 2 M GuHCl) to final ThT concentrations between 1 and 100 µM. ThT fluorescence intensity measurements were done as previously described.

### Seeded aggregation

Aliquots of sonicated fibril samples were added to monomeric MoPrP (89–230) solutions (0.5 mg/ml protein, 2 M GuHCl, 50 mM sodium phosphate, pH 6.0) with ThT to a final fibril/monomer ratio of 1:10 and ThT concentration of 100 µM. During aggregation, the samples were incubated at a stable 60 °C temperature. The aggregation reaction was observed using a Qiagen Rotor-Gene Q real-time analyzer^43^ for 1000 min, with measurements taken every minute.

### Fibril dissociation assay

Sonicated fibril samples were diluted to 20% of their initial concentration to a range of different concentration GuHCl solutions using 50 mM sodium phosphate buffers (pH 6.0) with and without 6 M GuHCl. The samples were incubated for 1 hour at 25 °C, then ThT was added to a final concentration of 100 µM. Measurements of ThT fluorescence were done as previously described.

### Atomic force microscopy

AFM images were acquired as described previously^44^. 20 µL of each sample was deposited on freshly cleaved mica and incubated for 1 minute. The samples were then rinsed with 1 mL of MilliQ water and dried under gentle airflow. AFM images were acquired using Dimension Icon (Bruker) atomic force microscope operating in tapping mode and equipped with a silicon cantilever RTESPA-300 (Bruker). All images were acquired at high-resolution (1024 × 1024 pixels). Three-dimensional AFM maps were flattened using SPIP or Gwyddion software. Height and width of the fibrils was determined from line profiles taken perpendicular to the fibril axes. Length was determined by tracing along the median axis of each aggregate.

### Fourier-transform infrared spectroscopy (FTIR)

MoPrP fibril samples were dialyzed into MilliQ water for 24 hours at 4 °C, then fibrils were separated from buffer solution by centrifugation at 20 000 x g for 30 min and resuspended in 1 mL of D_2_O, the centrifugation-resuspension procedure was repeated three times. All samples were then sonicated for 60 s (MS-72 tip, 20% sonication strength). The FTIR spectra were recorded as described previously^44^ using Bruker Vertex 80 v IR spectrometer equipped with mercury cadmium telluride (MCT) detector. For all measurements, CaF_2_ transmission windows and 0.05 mm Teflon spacers were used. Spectra were recorded at room temperature under near-vacuum conditions (~2 mBar). For each spectrum, 256 interferograms of 2 cm^−1^ resolution were co-added. A D_2_O spectrum was subtracted from each sample spectrum. All the spectra were normalized to the same area of amide I/I′ band (1700–1595 cm^−1^). All data processing was performed using GRAMS software.

## Results

**Figure 1:**
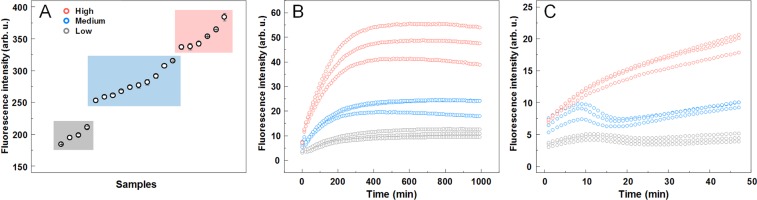
Separation of fibrils by ThT fluorescence intensity. (**A**) ThT fluorescence emission intensities of twenty MoPrP fibril samples prepared under identical conditions. The samples are grouped into three intensity regions, with some samples having low (grey), medium (blue) and high (red) emission intensities. (**B**) Seeded aggregation kinetics of the three intensity region fibrils using 10% of low, medium and high intensity fibrils. (**C**) Fluorescence intensity “drops” at the early stages of the aggregation process.

Twenty MoPrP samples were aggregated under the exact same conditions and examined by a ThT fluorescence assay, which yielded a very uneven distribution of intensity values (Fig. 1A). The samples were then separated into low (LI), medium (MI) and high (HI) intensity groups and combined for further studies.

### Seeding potential

All three groups were used in a seeded aggregation experiment to determine their aggregation kinetics. The results from all three samples presented both different fluorescence intensities, as well as distinct aggregation kinetic curves (Fig. 1B). The time needed for the fluorescence intensity to reach 50% of the maximum intensity value was 147 ± 11 min for LI, 108 ± 20 min for MI and 96 ± 6 min for HI samples. The fluorescence values at the end of the reaction had a similar intensity distribution (low, medium and high) as the initial samples (Fig. 1B). The MI and LI fibril seeding kinetic curves had an unusual “drop” at the early stages of the reaction (Fig. 1C), which is most evident in the case of MI fibrils.

### ThT binding affinity

**Figure 2:**
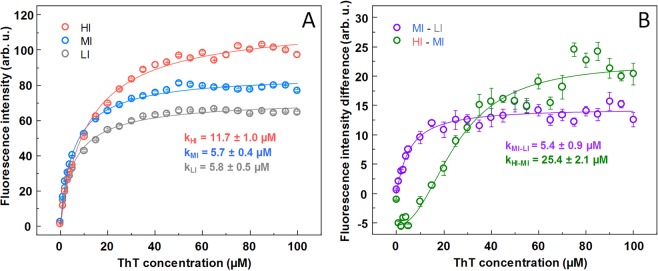
ThT binding to MoPrP amyloid fibrils probed by fluorescence assay. (**A**) ThT fluorescence emission intensity dependence on the concentration of ThT added to each fibril sample. (**B**) ThT fluorescence intensity differences between MI and LI samples and HI and MI samples. Hill equation fitting was done to determine the ThT concentration (k) at which the signal intensity midpoint is reached.

The fibril samples were examined for their ThT binding affinity by mixing the fibrils with a range of different ThT concentrations. We can see that in the case of both MI and LI fibrils (Fig. 2A), while the intensity of ThT fluorescence emissions is different, the ThT concentration at which the signal intensity midpoint is reached is within margin of error (5.8 ± 0.5 µM for LI and 5.7 ± 0.4 µM for MI). However, this value is considerably higher (11.7 ± 1.0 µM) when examining the ThT binding affinity of fibrils in the HI sample (Fig. 2A). Subtracting the LI sample intensities from MI shows that the intensity midpoint ThT concentration of the resulting curve (Fig. 2B) is similar to both LI and MI values (5.4 ± 0.9 µM) seen in Fig. 2A, suggesting the higher intensity is the result of more ThT molecules bound in a similar mode. However, subtracting MI sample intensities from HI results in a completely different curve (Fig. 2B) with a much higher midpoint value (25.4 ± 2.1 µM). The signal intensity difference is also negative at low ThT values, suggesting that less ThT binds in the LI or MI mode, while more binds in a mode not present in the other two types of fibrils.

### Fibril stability and secondary structure

**Figure 3:**
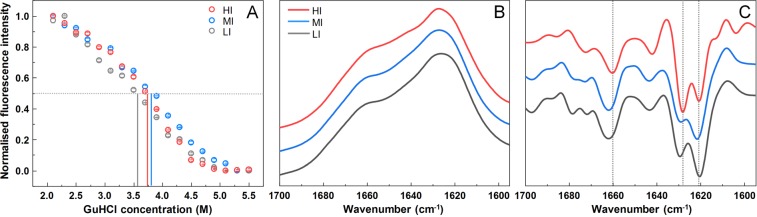
Dissociation assay of HI, MI, and LI fibrils and comparison of their secondary structures. (**A**) Normalized ThT fluorescence intensity values at different GuHCl concentrations, where the grey dotted line represents 50% of normalized fluorescence intensity and coloured lines correspond to each sample’s GuHCl concentration at which the 50% intensity value is reached. (**B**) FTIR spectra of fibril samples and second order derivative spectra (**C**), where grey dotted lines show wavenumbers at which the differences between spectra can be observed.

Fibril dissociation under denaturing conditions was measured to determine possible structural differences between the three samples. There is a slight variation in the dissociation midpoint values (3.6 M for LI, 3.7 M for HI and 3.8 M for MI fibrils) when comparing normalized dissociation curves (Fig. 3A), however, such a minor difference does not constitute any substantial stability variations between the samples. This indicates that the formed aggregates do not have strain-specific structural stability. PK-digestion of all three samples also shows that there are no substantial variations in the size of the PK-resistant core (Fig. S3). In order to further examine the structure of these fibrils, FTIR spectra of all three samples were recorded.

The LI and MI sample fibrils appear to have a very similar FTIR spectra (Fig. 3B,C) in the amide I/I’ region, both exhibit maxima at ~1627 (with the main minimum of the second derivative at ~1621 cm^−1^ and a weaker one at ~1629 cm^−1^) and a shoulder which is reflected by the minimum of the second derivative at ~1662 cm^−1^. However, there is a noticeable difference between them and the HI fibril sample, which exhibits a maximum at ~1627 (with the main minimum of the second derivative at ~1628 cm^−1^ and a weaker one at ~1621 cm^−1^) and a shoulder which is reflected by the minimum of the second derivative at ~1660 cm^−1^. (Fig. 3B,C). In all three cases, the band’s shape and position are characteristic for the amyloid’s parallel beta-sheet structure^45^. But the dramatic reversal of the relative intensities of the two spectral components around 1621 and 1628–29 cm^−1^ in the second derivative spectra (Fig. 3C) of LI vs. HI fibrils confirms the difference between these aggregates in the level of secondary structure.

### Fibril morphology

**Figure 4:**
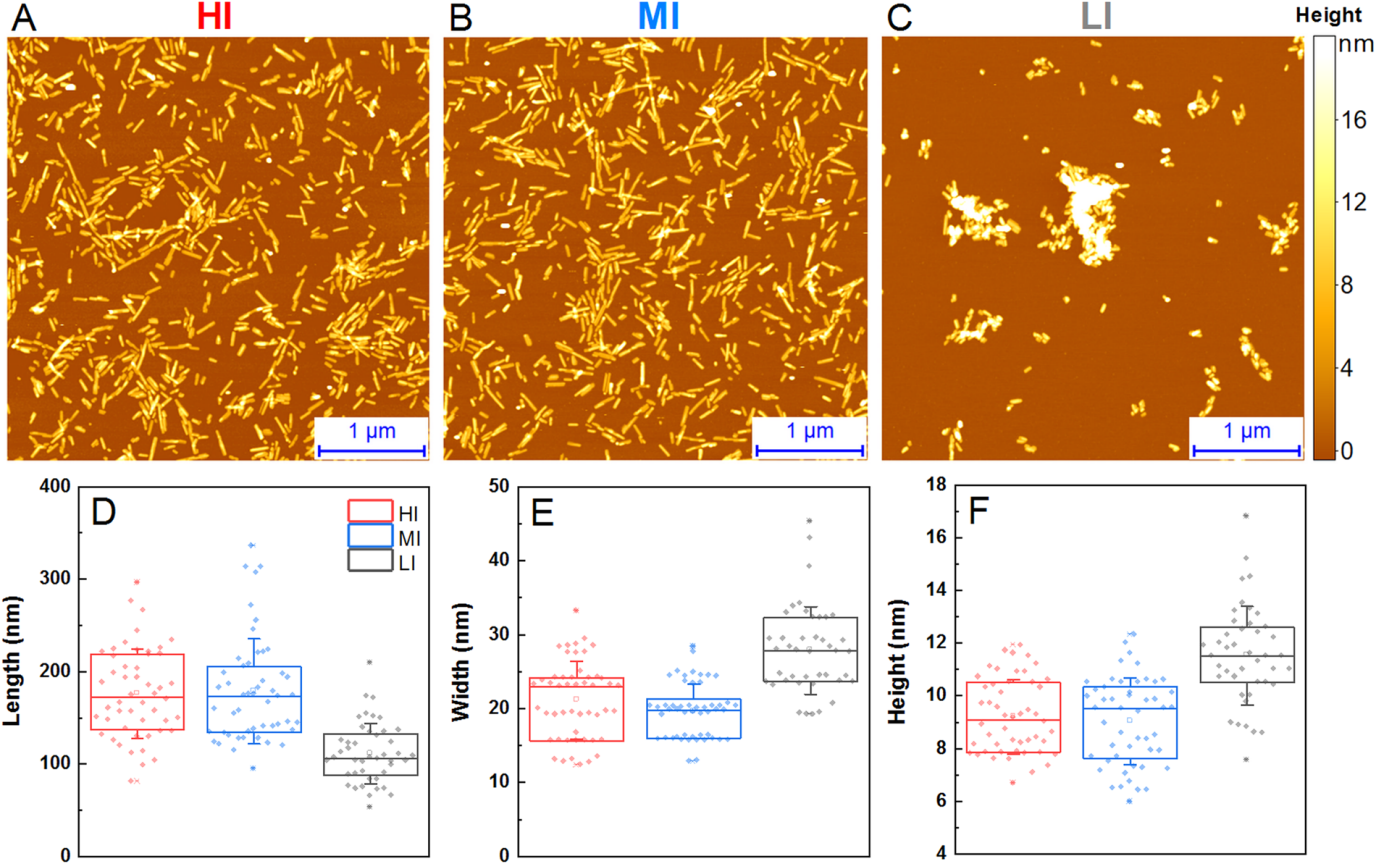
Atomic force microscopy of fibril samples and aggregate size distribution. Images of high intensity (**A**), medium intensity (**B**) and low intensity (**C**) fibrils. Length (**D**), width (**E**) and height (**F**) of single fibrils, where the box plot indicates the interquartile range, error bars are for one standard deviation.

The fibrils from each sample were examined by atomic force microscopy to determine if there are any visible structural differences between them. The AFM images show small and completely dispersed fibril fragments in the case of HI (Fig. 4A) and MI (Fig. 4B) samples. Conversely, the LI (Fig. 4C) sample contains much larger aggregate clusters. Examining the length (Fig. 4D), width (Fig. 4E) and height (Fig. 4F) of single fibrils reveals that the LI aggregates are significantly shorter (~100 nm), wider (~27 nm) and higher (~11 nm), when compared to both MI and HI fibrils, which have a length of ~175 nm, width of ~20 nm and height of ~9 nm. The surface of all samples appears to be relatively even, with no visible periodicity or twistedness (Fig. S4). Sample sonication has an insignificant effect on the dispersion of these aggregates, which indicates that the LI fibrils are highly prone to self-association and quickly reassemble into large clusters (Fig. S5).

## Discussion

Comparison of all results leaves no doubts that LI and HI samples represent distinct amyloid fibril conformations. Besides almost a double difference in ThT fluorescence intensity (Fig. 1A), which gets even bigger in reseeded samples (Fig. 1B), different kinetic profiles of HI and LI fibril self-replication (Fig. 1B,C) were observed (ThT intensity drop at early stages of aggregation when LI fibrils were used as seeds, and no drop in case of HI seeds). Different major minima in FTIR second derivative suggests that two different populations of intra-fibrillar beta-strands are dominant in the structure of HI and LI fibrils (Fig. 3C), similar as were earlier observed in environment-induced polymorphism of amyloid fibrils^46^. Distinct conformations of HI and LI aggregates are also supported by the differences in ThT binding (Fig. 2A,B). Finally, in the AFM images (Fig. 4A–C) we can see that the LI fibrils are shorter, wider and higher than HI fibrils, and they are clumped together, as opposed to the HI sample. Such cluster formation was even observed by a simple visual inspection, with HI samples being almost completely clear and LI having cloudy precipitates. In fact, the HI fibrils were so dispersed, that their centrifugation had to be carried out after dialysis into Milli-Q water, in order to improve the rate of sedimentation. While the LI fibrils quickly associate back into such clusters (Fig. S5) even after sonication, which suggests that LI aggregates have different surface properties than HI aggregates.

The case of MI sample is less clear. Medium ThT fluorescence (Fig. 1A) may arise if MI samples would contain a mixture of LI and HI fibrils. Kinetic profiles of fibril self-replication (Fig. 1B,C) would also fit within the mixture hypothesis. FTIR spectral properties of MI samples are similar to LI (Fig. 3B,C), however there are some minor differences in peak positions and ratios between the minima of second derivative, so the possibility of a mixture cannot be completely excluded, but LI fibrils must be the major component of the mixture. ThT binding data also points to the similarities between LI an MI fibrils (Fig. 2A,B), but this experiment is not very precise, so we cannot completely reject the possibility of a mixture with the amount of LI aggregates several times higher than HI. But the AFM data shows opposite results (Fig. 4A–C). The appearance, length, width and height of MI fibrils are different from LI fibrils (Fig. 4D–F) but are very similar to HI fibrils. It means that the idea of the MI sample as a mixture of HI an LI aggregates may not be accurate. An alternative hypothesis would claim MI as an independent conformation of amyloid aggregates, different from both LI and HI conformations. In order to clear up the confusion regarding the MI sample, two additional aggregation experiments were carried out, using prion proteins from different purification batches (Fig. S6). In both cases, the difference between LI and HI was quite obvious in both the AFM (Fig. S7) and FTIR (Fig. S8) data, however, in one case, the MI sample was similar to LI and in another – to HI. This means that the intermediate samples are not composed of a different fibril conformation and are likely mixtures with varying degrees of LI and HI.

One can think of how different aggregate conformations may form under the same experimental conditions. According to the nucleated polymerization model, fibril formation starts from nucleation. To form a nucleus, a group of soluble protein molecules must get together and misfold into an amyloid structure. Once the nucleus is formed, it can rapidly grow into fibrils by capturing and refolding protein molecules from the solution. The number of fibrils can grow either via formation of new nuclei, or via fragmentation of the existing fibrils. Amyloid nucleation is a stochastic process^47^, so if the protein can misfold into several different amyloid conformations, then it is probable that the structure of the first nucleus formed in one tube will be different from one in another tube. Fibril elongation rate is much higher than the nucleation rate, so once the first nucleus is formed, it can grow into a long fibril and, due to vigorous shaking, get fragmented into many short fibrils before the second nucleus is formed. In case of such scenario, once all protein in the tube gets aggregated, the majority of amyloid fibrils will have the same conformation as the first nucleus, and the stochastic nature of nucleation can be the reason for polymorphism of amyloid fibrils formed under identical conditions.

## Conclusions

Our findings confirm that prion protein can misfold into at least two distinct amyloid conformations even under identical conditions and can be quickly distinguished by comparing ThT fluorescence emission intensities. Such stochastic polymorphism of amyloid fibrils may be the reason for low reproducibility in amyloid research. A quality-control of each sample by the comparison of ThT intensity could help to improve it.

## Supplementary information

Supplementary information.

## Data Availability

The datasets generated during and/or analysed during the current study are available from the corresponding author on reasonable request.
