# Supplementary material for: Formation of distinct prion protein amyloid fibrils under identical experimental conditions
Source: Sci Rep. 2020 Mar 12;10:4572. doi: 10.1038/s41598-020-61663-2 (PMC7067779; doi:10.1038/s41598-020-61663-2)
Supplement: Supplementary file 1 — Supplementary information. [file 41598_2020_61663_MOESM1_ESM.pdf]

Formation of distinct prion protein amyloid fibrils under identical experimental conditions

Mantas Ziaunys, Tomas Sneideris, Vytautas Smirnovas*

Institute of Biotechnology, Life Sciences Center, Vilnius University, Vilnius, Lithuania

*Corresponding Author:

Vytautas Smirnovas

Sauletekio al. 7, Vilnius, LT-10257, Lithuania

Email address: vytautas.smirnovas@bti.vu.lt

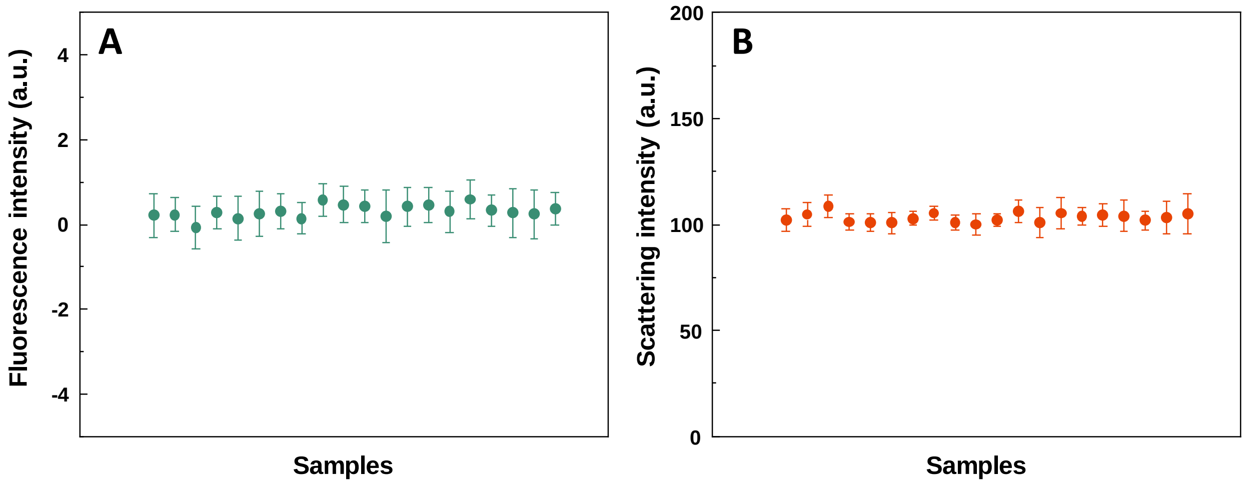

Supplementary Figure S1. ThT fluorescence (A) and light scattering (B) intensity distribution of 20 MoPrP fibril samples. ThT fluorescence was measured using 440 nm excitation and 480 nm emission wavelengths and the intensity of a solution without protein was subtracted from each value. Right-angle light scattering was measured using a 600 nm excitation and emission wavelength. For each sample, three measurements were averaged.

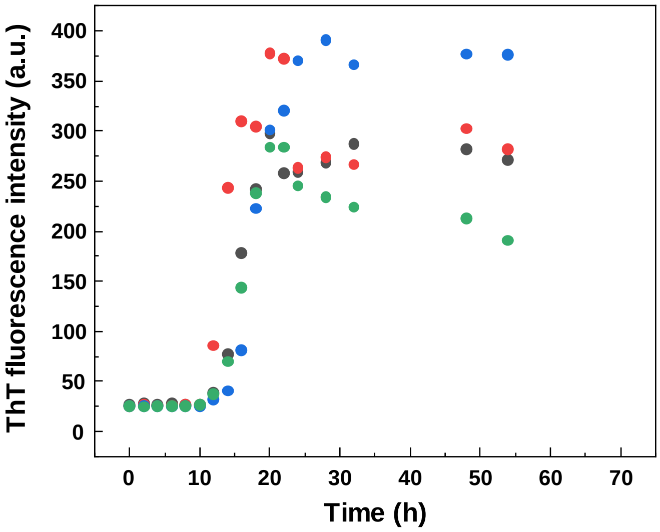

Supplementary Figure S2. Spontaneous aggregation of MoPrP89-230 (0.5 mg/ml protein concentration, 2 M GuHCl, 50 mM phosphate buffer (pH 6.0) at constant agitation (220 RPM) and temperature (37 °C)). ThT fluorescence intensity was tracked by taking aliquots of each sample at different time points. Measurements were performed as described in the method section.

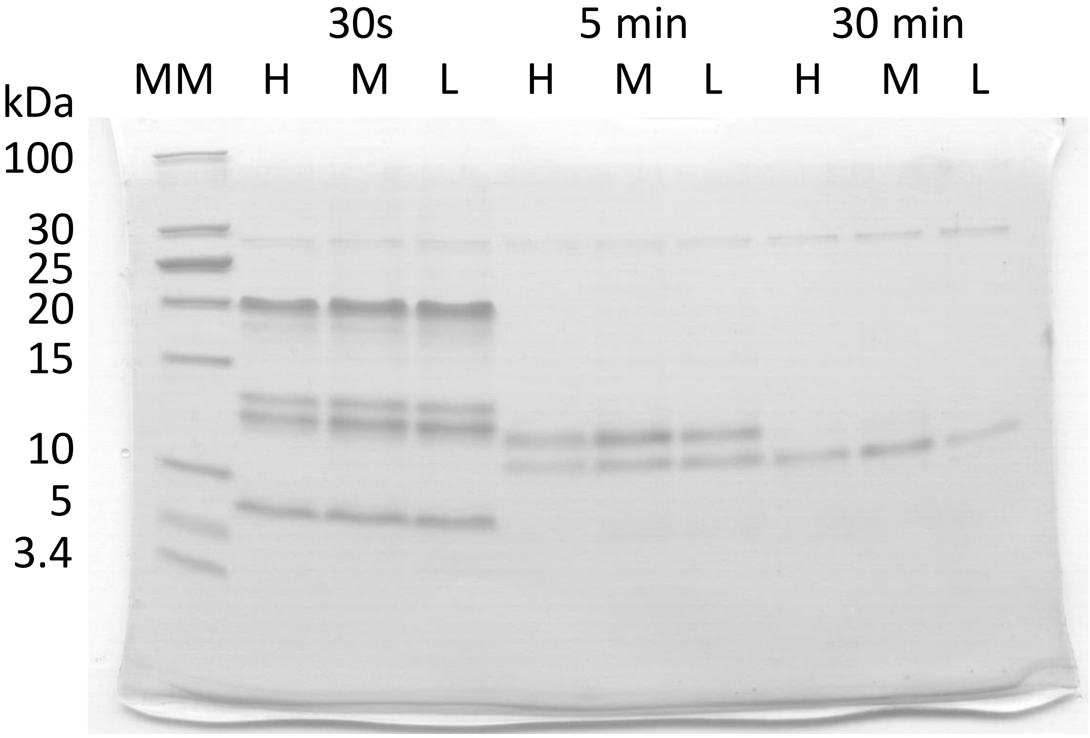

Supplementary Figure S3. Fibril samples (0.5 mg/ml) were centrifuged at 10’000 x g for 30 min. Subsequently, fibrils were resuspended in 50 mM Tris buffer solution (pH 8.0) and centrifuged again. Then fibrils were resuspended in 200 µl of Tris buffer solution and sonicated for 30 s using Bandelin Sonopuls ultrasonic homogenizer equipped with a MS 72 tip (20 % amplitude). After sonication, the fibril solution was supplemented with 2.5 µl of 4 mg/ml Proteinase K and incubated for 30 s, 5 min and 30 min at 37 °C with 600 RPM agitation in a Ditabis thermomixer MHR 23. 17 µl of each sample was collected, supplemented with 3 µl of 10 mM PMSF, 96% EtOH and 20 µl of 2 X SDS-Page sample buffer containing 6 M of urea. Samples were heated for 15 min at 98 °C and subsequently analysed via Tricine–SDS-PAGE [1]. The high, medium and low intensity fibril samples are indicated by H, M and L letters respectively.

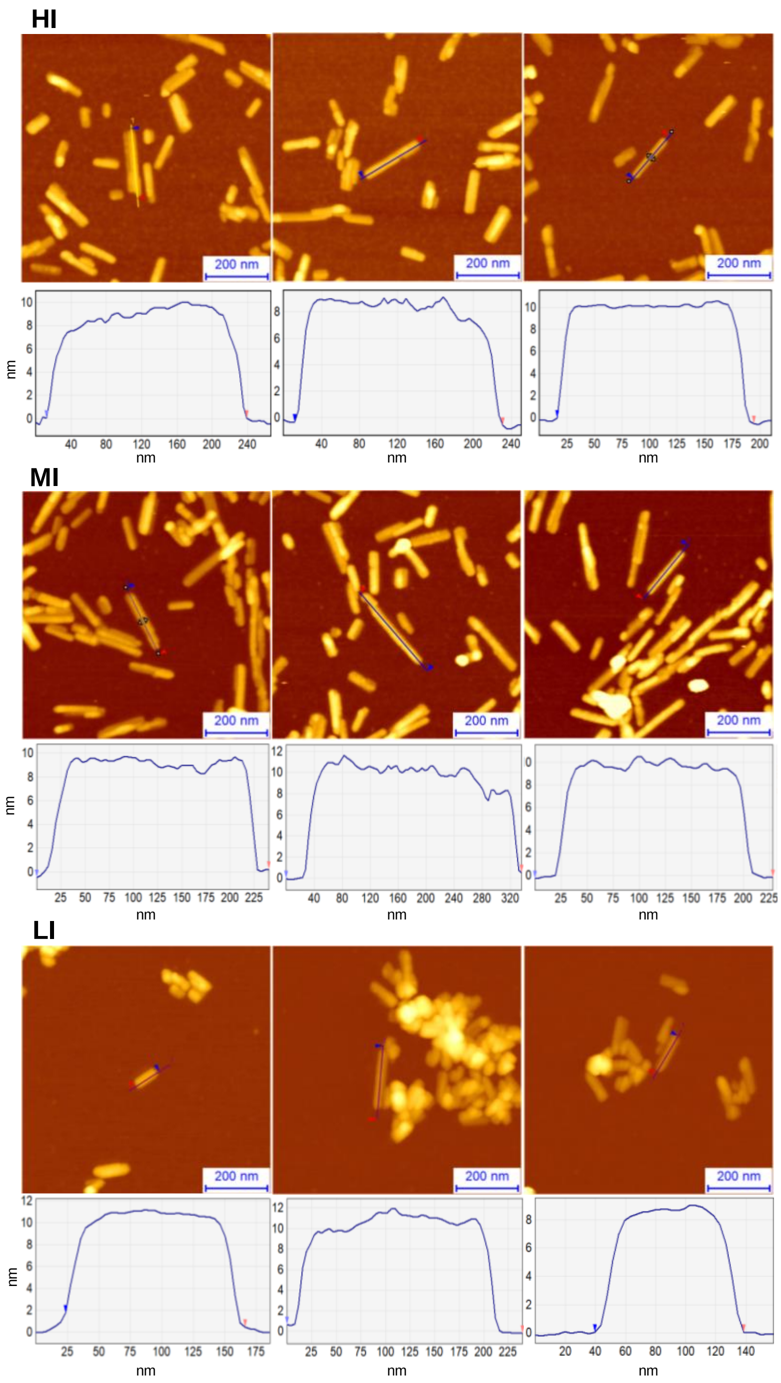

Supplementary Figure S4. LI, MI and HI sample fibril surface analysis by AFM. Fibril height was traced along the fibril's axis for three randomly selected aggregates in LI, MI and HI samples.

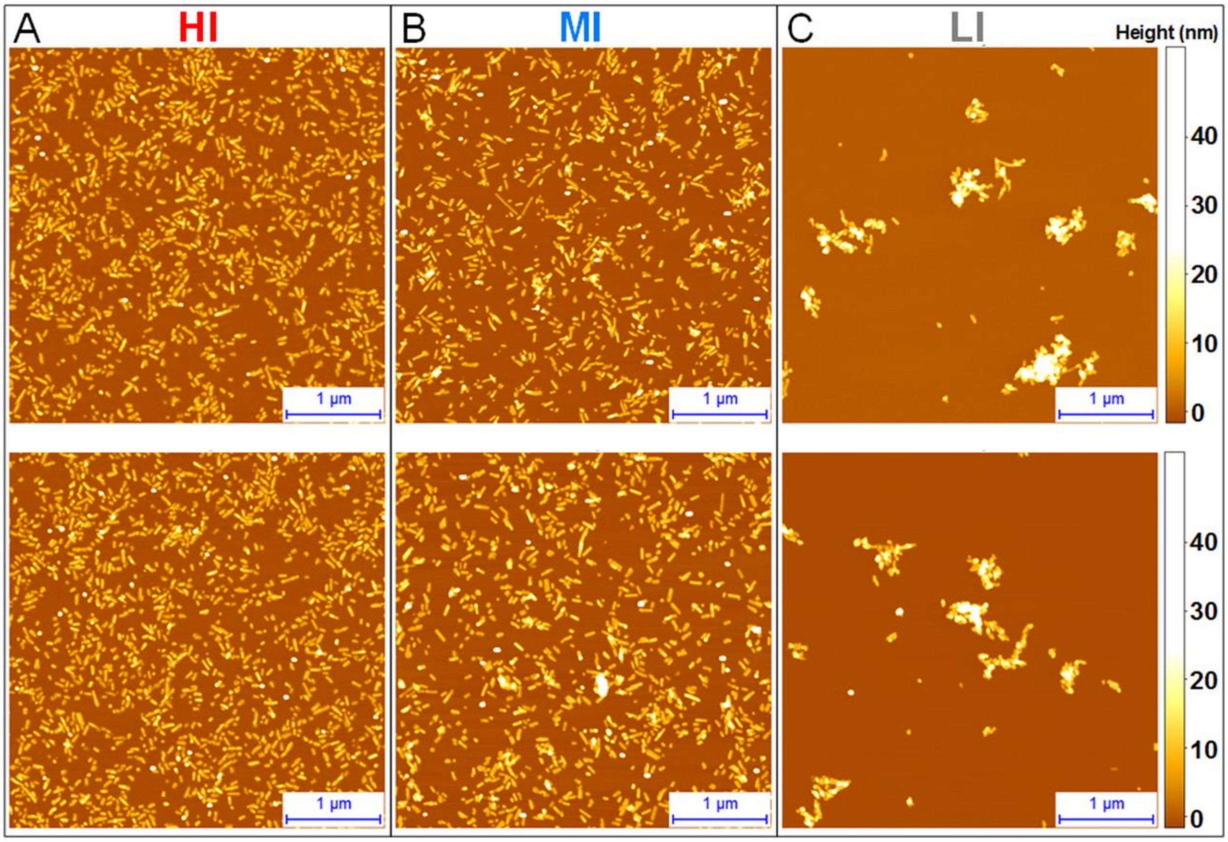

Supplementary Figure S5. AFM images of LI, MI and HI samples after sonication.

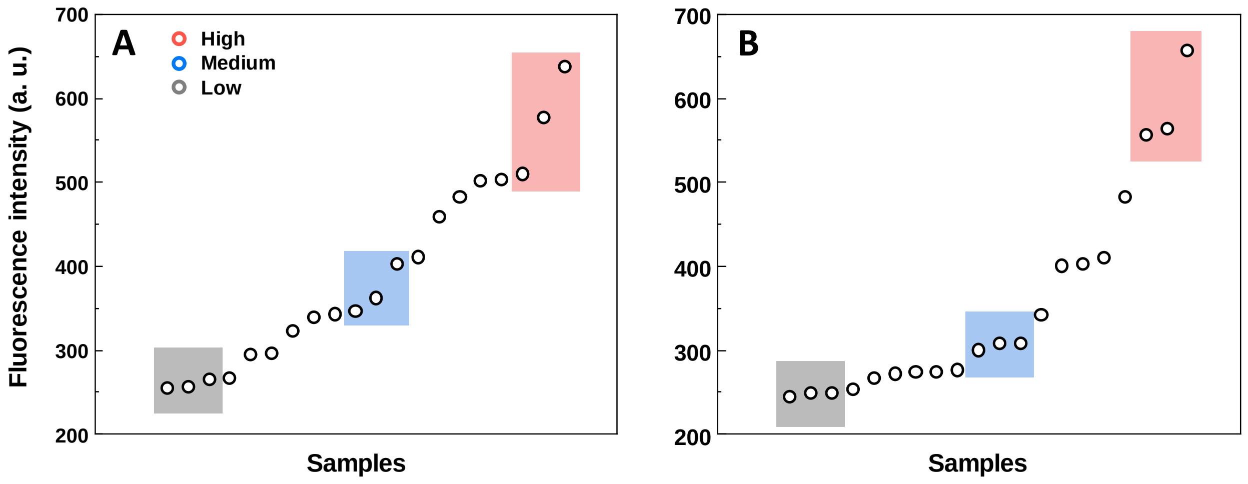

Supplementary Figure S6. ThT fluorescence emission intensities of twenty MoPrP fibril samples prepared under identical conditions from two different protein batches (A, B). The samples are grouped into three intensity regions, with some samples having low (grey), medium (blue) and high (red) emission intensities.

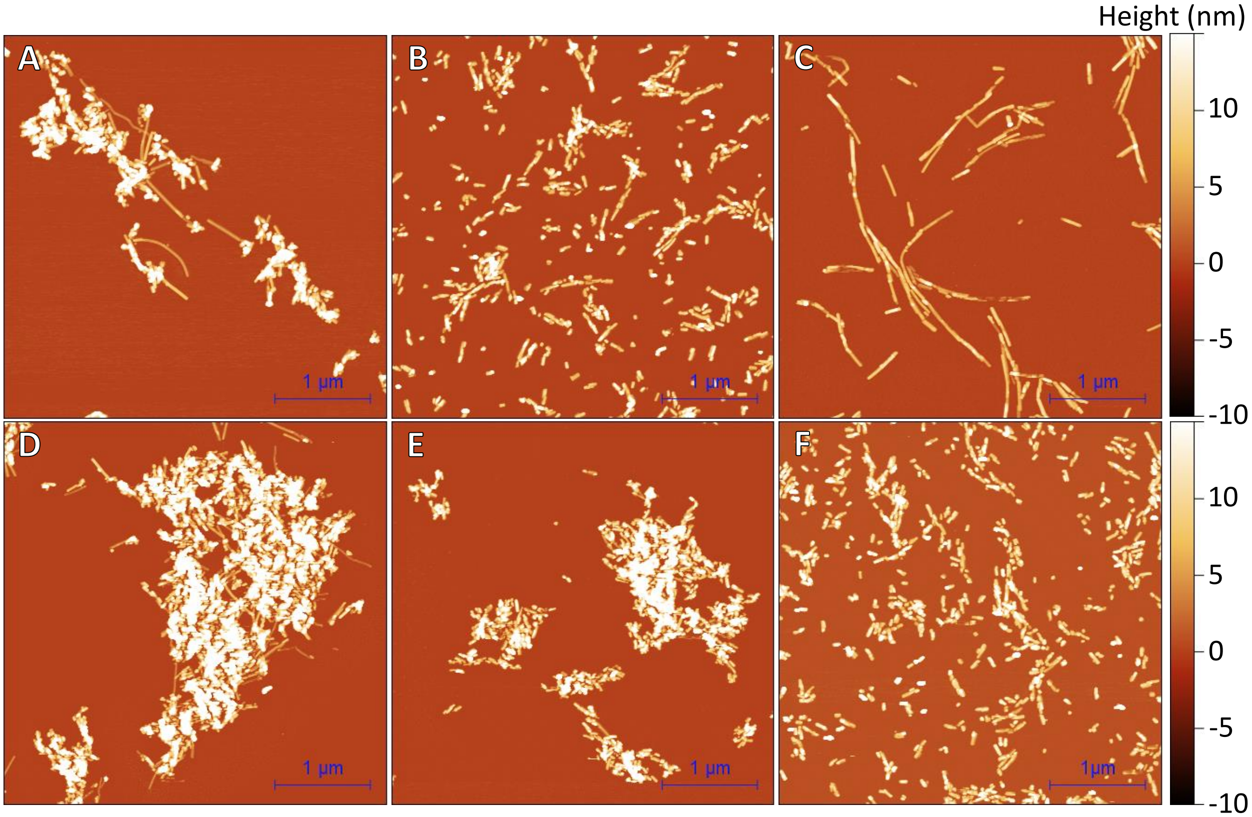

Supplementary Figure S7. AFM images of LI (A, D), MI (B, E) and HI (C, F) fibril samples prepared from different protein batches.

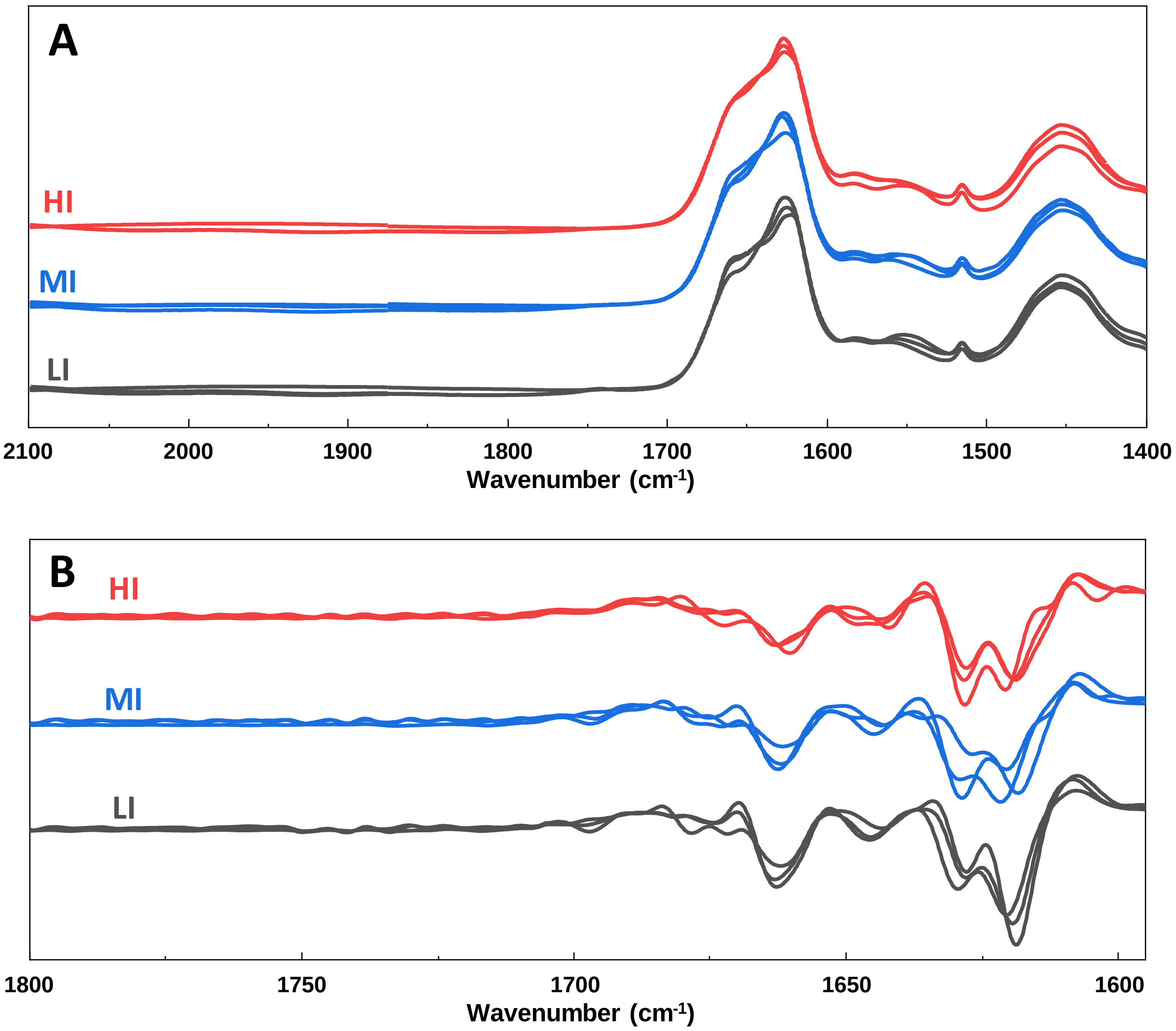

Supplementary Figure S8. FTIR spectra (A) and second derivatives (B) of LI, MI and HI fibril samples from three different protein batches.

References

1. Schägger, H. Tricine–SDS-PAGE. *Nat. Protoc.* **1**, 16–22 (2006).
